# Color Stabilization of Apulian Red Wines through the Sequential Inoculation of *Starmerella bacillaris* and *Saccharomyces cerevisiae*

**DOI:** 10.3390/molecules26040907

**Published:** 2021-02-09

**Authors:** Matteo Velenosi, Pasquale Crupi, Rocco Perniola, Antonio Domenico Marsico, Antonella Salerno, Hervè Alexandre, Nicoletta Archidiacono, Mario Ventura, Maria Francesca Cardone

**Affiliations:** 1 Consiglio per la Ricerca in Agricoltura e L’analisi Dell’economia Agraria-Centro di Ricerca Viticoltura ed Enologia (CREA-VE), Turi, 148-70010 Bari, Via Casamassima, Italy; matteo.velenosi@tiscali.it (M.V.); pasquale.crupi@crea.gov.it (P.C.); rocco.perniola@crea.gov.it (R.P.); adomenico.marsico@crea.gov.it (A.D.M.); antonellasalerno10@gmail.com (A.S.); 2 Dipartimento di Biologia, Università degli Studi di Bari Aldo Moro, 4-70124 Bari, Via Orabona, Italy; nicoletta.archidiacono@uniba.it; 3 UMRProcédésAlimentaires et Microbiologiques, Equipe VAlMiS (Vin, Aliment, Microbiologie, Stress), AgroSupDijon—Université de Bourgogne/Franche-Comté, IUVV, Rue Claude Ladrey, BP 27877, 21000 Dijon, France; rvalex@u-bourgogne.fr

**Keywords:** HPLC-UV-ESI-MSn, free anthocyanins, co-pigmented anthocyanins, mixed fermentation, *starmerella bacillaris*, PCA

## Abstract

Mixed fermentation using *Starmerella bacillaris* and *Saccharomyces cerevisiae* has gained attention in recent years due to their ability to modulate the qualitative parameters of enological interest, such as the color intensity and stability of wine. In this study, three of the most important red Apulian varieties were fermented through two pure inoculations of *Saccharomyces cerevisiae* strains or the sequential inoculation of *Saccharomyces cerevisiae* after 48 h from *Starmerella bacillaris*. The evolution of anthocyanin profiles and chromatic characteristics were determined in the produced wines at draining off and after 18 months of bottle aging in order to assess the impact of the different fermentation protocols on the potential color stabilization and shelf-life. The chemical composition analysis showed titratable acidity and ethanol content exhibiting marked differences among wines after fermentation and aging. The 48 h inoculation delay produced wines with higher values of color intensity and color stability. This was ascribed to the increased presence of compounds, such as stable A-type vitisins and reddish/violet ethylidene-bridge flavonol-anthocyanin adducts, in the mixed fermentation. Our results proved that the sequential fermentation of *Starmerella bacillaris* and *Saccharomyces cerevisiae* could enhance the chromatic profile as well as the stability of the red wines, thus improving their organoleptic quality.

## 1. Introduction

Yeast metabolism, during the winemaking process, influences the wine organoleptic properties and, consequently, wine quality. It can directly or indirectly affect the content of several compounds related to both the aroma and color characteristics. Recent studies on mixed starter cultures have proved that the resulting wines differ significantly, concerning both their chemical composition and sensory characteristics. Different yeast species and the ratio of non-*Saccharomyces/Saccharomyces* yeasts determine the organoleptic properties of the final product, and therefore contribute differently to the improvement or depreciations of wine quality [1].

The color is the most important visual attribute of red wines [2], which strongly impresses consumers’ purchasing preference [3]. Moreover, it influences the perception of other sensory properties, such as aroma and flavor. Therefore, winemakers have accustomed to adopting suitable practices that improve color extraction and enhance the stability of chromatic characteristics of wine over time [4]. The color of red wines is mainly due to anthocyanins, which are transferred from grape skins into wine throughout the maceration/fermentation process [5]. Whereas, the stability of color during wine aging is affected by the phenolic derivatives which stabilize anthocyanins through co-pigmentation reactions [6,7]. The types and concentrations of polyphenols in wine may depend on the grape variety, the degree of ripening [8], and the vine growing methods employed, specifically the pruning and training system [7,9]. The joining of additives (i.e., enzymes, yeasts, or tannins) during winemaking is also a determinant [4,10,11].

In this aspect, there has been growing interest in the use of non-*Saccharomyces* yeasts due to the positive impact some of their metabolites exert on wine quality [12,13]. Many authors have demonstrated that non-*Saccharomyces* yeasts have a protective effect on wine color [4,10,14]. Among these, *Starmerella bacillaris (S. bacillaris)* [15] has been considered one of the most promising non-*Saccharomyces* yeasts [16,17,18] (having strong fructophilicity, high tolerance to low temperatures, and ability to grow at an elevated sugar concentration) [19]. However, non-*Saccharomyces* yeasts possess low fermentation ability and cannot carry out the must fermentation alone, due to their ethanol sensitivity [20,21]. Consequently, their use in combination with selected *Saccharomyces cerevisiae (S. cerevisiae)* (Desm. Meyen 1838) strains is necessary for completing the fermentation and taking advantage of their unique features [22].

Recently, a meaningful knowledge has been accumulated about the importance of yeast inoculation density, timing, and combination of strains in improving the organoleptic properties of wines [16,23,24]. The use of *S. bacillaris* during winemaking has allowed increasing the must total acidity and enhancing the color intensity of wine [25,26]. Similarly, this yeast strain has led to a higher production of pyruvic acid, which is involved in the formation of stable pigments (i.e., vitisin A and B), compared to *Saccharomyces* [27]. Thereby, it could be hypothesized that a mixed fermentation (by employing both the yeasts, sequentially) works in improving the color intensity as well as the color stability of wine. This study aimed at comparing the anthocyanin profiles and chromatic characteristics of wines produced through two mono-*S. cerevisiae* fermentations (SCE16 and SCE138, respectively) or the sequential fermentation of *S. bacillaris* and *S. cerevisiae* SCE16/SCE138 inoculated 48 h later. The analyses were conducted on wines produced from the most important red Apulian varieties (Primitivo, Negramaro, and Aleatico) at draining off and after 18 months of bottle aging, to investigate the potential of the color stabilization and shelf life of these wines.

## 2. Results

### 2.1. Interaction between Saccharomyces Yeast Strains and Pilot Scale Fermentation

In order to evaluate the suitability of the three yeast strains in mixed fermentation, we first evaluated the phytotoxic activity towards each other both on plate and liquid culture assays.

In the experiment performed on the plate assay, the three yeast strains were able to grow independently of the previous growth of the other tested yeast strain on the cellophane disc. Furthermore, the growth curves of each yeast strain are similar regardless of the type of filtered supernatant added (Supplementary Figure S1). Likewise, no inhibition of growth was observed in the liquid culture assay combining two yeast strains together, both considering the interaction of *S. cerevisiae* strains or *S. bacillaris* with each of the *S. cerevisiae* strain. Taking into consideration the absence of any phytoxic activity among the different combinations of yeast strains, we were able to test their effect on wine production in a mixed fermentation where the two *S. cerevisiae* strains (SCE16 and SCE138, 1:1) were added together 48 h after the inoculation of *S. bacillaris* (FA18), and compare this trial with mono-*saccharomyces* fermentation. Moreover, in order to assess the fermentation ability of the chosen yeast combination with respect to mono-fermentation and, in particular, to further verify the absence of any negative interaction in mixed fermentation among the yeast strains, fermentation kinetics were followed for each trial (Supplementary Figure S2). Mono inoculation SCE16 and SC138 showed a similar or equal consumption in sugar level in every variety considered, thus demonstrating the same fermentation capacity of the two *S. cerevisiae* strains. On the contrary, the mixed FA18 was characterized by a slow start, regarding the sugar consumption, reaching up to 7% in Primitivo, 8% in Negroamaro, and 13% in Aleatico. The higher delay we found in the Primitivo could be ascribed to the sugar concentration effect on the *S. bacillaris* activity, as previously described [28]. Indeed, the sugar and nitrogen composition of the grape must are key factors for the evolution of the alcoholic fermentation and the development of the yeasts [29,30]. Notably, also in the FA18 mixed fermentation, complete sugar consumption was reached around 4 days after the inoculum with the two *S. cerevisiae* strains, thus confirming the absence of a negative interaction between the yeast strains both considering *S. bacillaris* against *S. cerevisiae*, and between the two *S. cerevisiae* strains.

### 2.2. Basic Oenological Parameters and Chemical Composition

The chemical composition of Primitivo, Negramaro, and Aleatico wines produced by pure and mixed culture fermentation at draining off and after 18 months of bottle aging were listed in Table 1.

Overall, the fermentation type factor influenced the titratable acidity (A) of the wines. Indeed, samples obtained by mixed fermentation generally contained more acids, in particular, Negramaro and Aleatico FA18 wines had a significantly higher A (*p* < 0.01). These differences (ranging from 0.25 to 0.57 g/L) cannot be imputed to the main organic acids (citric, malic, tartaric, and lactic acids) whose values did not significantly change in all the wines (Supplementary Table S1).

Furthermore, pH values were not affected by the different fermentation protocols at drying off (Table 1). Conversely, these findings may be due to the capability of *S. bacillaris* strains to relatively synthesize high concentrations of keto acids either during the early stages of fermentation from sugar metabolism or from the corresponding amino acids (alanine for pyruvic acid and glutamate for α-keto glutaric acid), as previously reported [27,31,32]. On the contrary, we revealed a significantly higher pH value in 18 months aged wines connected to the partial tartaric precipitation that happened during aging in the bottle. However, not surprisingly, the slight decrease of A (total acidity) during the wines aging could also be due to a series of maturation reactions involving pyruvic acid [7].

No significant difference in the alcoholic degree (% *v*/*v*) was registered between pure and mixed fermentation in all the samples (Table 1). Moreover, the volatile acidity was strongly influenced by the fermentation protocol and bottle aging, as well as by the interaction of the two factors (*p* < 0.001), even though all the wines contained <0.40 g/L (Table 1), which cannot be considered detrimental to the sensorial quality of wine as in agreement with literature data [33]. Furthermore, we analyzed the polyphenolic content and we found that monomeric anthocyanins (MA), total anthocyanins (TA), and total polyphenols (TP) values appeared significantly higher in SCE16 and SCE138 than in the FA18 samples, especially for Negramaro and Aleatico (Table 1). Moreover, we detected a decrease of phenolics after 18 months which, was generally more marked in FA18 than SCE16 and SCE138 wines (Table 1).

### 2.3. HPLC-MS Analysis of Anthocyanin Profile in the Wines

The color changes during wine maturation are usually attributed to anthocyanin polymerization reactions and the evolution of co-pigments resulting from interactions between anthocyanins and other compounds at the fermentation phase [34,35]. For these reasons, we investigated the anthocyanin profile of the wines by HPLC-MS analyses and the pigments, identified through their retention time (RT), molecular ion (M^+^), and principal MS/MS fragments, as listed in Table 2.

Five mono glucoside anthocyanins, namely delphinidin (3), cyanidin (5), petunidin (6), peonidin (8), and malvidin (9), together with malvidin-3-*O*-acetylglucoside (15), malvidin-3-*O*-caffeoylglucoside (17), cyanidin-3-*O*-(*p*-coumaroyl)glucoside (17), peonidin-3-*O*-trans-(*p*-coumaroyl)glucoside (20), and malvidin-3-*O*-trans-(*p*-coumaroyl)glucoside (21) were revealed in all the samples. Whilst other acyl compounds, such as peonidin-3-*O*-acetylglucoside (14), petunidin-3-*O*-(*p*-coumaroyl)glucoside (18), and malvidin-3-*O*-*cis*-(*p*-coumaroyl)glucoside (18), also belonging to the group of free-anthocyanins directly extracted from grape skin [36,37], were not detected in Aleatico wines. Four compounds corresponding to carboxy-pyranoanthocyanins derived from the reaction between glucoside anthocyanins and pyruvic acid (A-type vitisins) were also identified (Table 2). In particular, petunidin (7) and malvidin (10) 3-*O*-glucoside pyruvate were present in all the samples, while peonidin (13) and malvidin (14) 3-*O*-(*p*-coumaroyl)glucoside pyruvate were absent in Aleatico wines. Two well resolved chromatographic peaks (11 and 12) referring to isobaric ions with similar MS/MS spectra were achieved for the species with [M]+ at *m*/*z* 809, which were identified as isomers of malvidin-3-*O*-glucoside-8-ethyl-(epi)catechin [38]. Then, other ethylidene-bridged flavanol anthocyanins, namely peonidin-3-*O*-(*p*-coumaroyl)glucoside-8-ethyl-(epi)catechin (19) and malvidin-3-*O*-(*p*-coumaroyl)glucoside-8-ethyl-(epi)catechin (23), were revealed in the wines (Table 2). With regards to vinyl-linked flavanol anthocyanins, also known as flavanol pyranoanthocyanins [33], malvidin-3-*O*-acetylglucoside-4-vinyl-(epi)catechin (16) and malvidin-3-*O*-glucoside-4-vinyl-(epi)catechin (22) were only detected in Primitivo and Negramaro wines, respectively. Finally, three flavanol-anthocyanins derivatives, having molecular ions and fragmentation patterns typical of (epi)-catechin-peonidin (1) or malvidin-3-*O*-glucoside (2 and 4) adducts [7] were found (Table 2).

**Figure 1:**
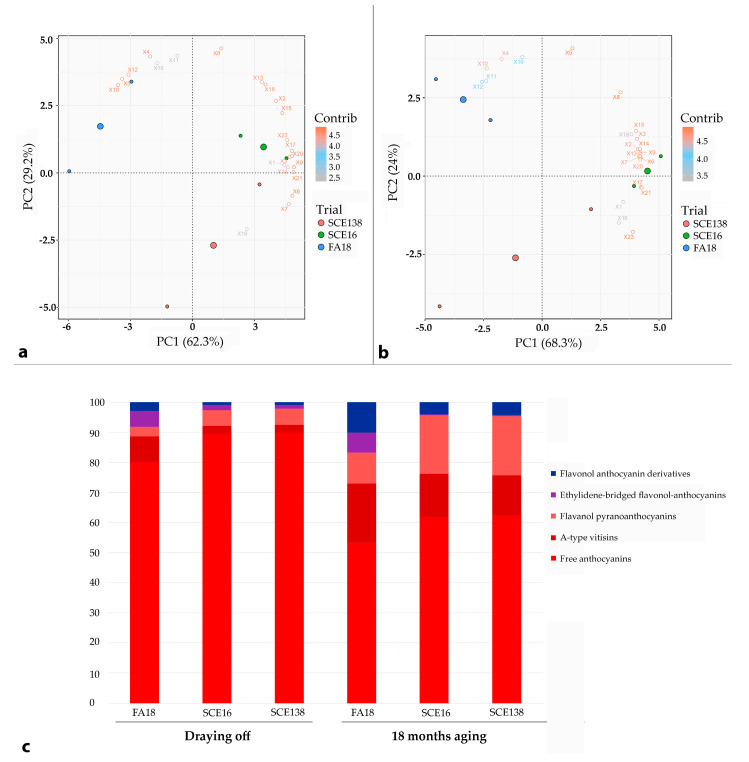
PCA—Primitivo. Principal component diagram of anthocynin-derived red pigments in Primitivo wines SCE138 (green point), SCE 16 (blue point), FA18 (red point), and distribution (percentage) calculated (**a**) at draining off and (**b**) after 18 months of bottle aging; in (**c**), we report the percentage of each pigment as measured by HLPC assays both at draining off and after 18 months of bottle aging. Variables correspond to peaks reported in Table 2.

**Figure 2:**
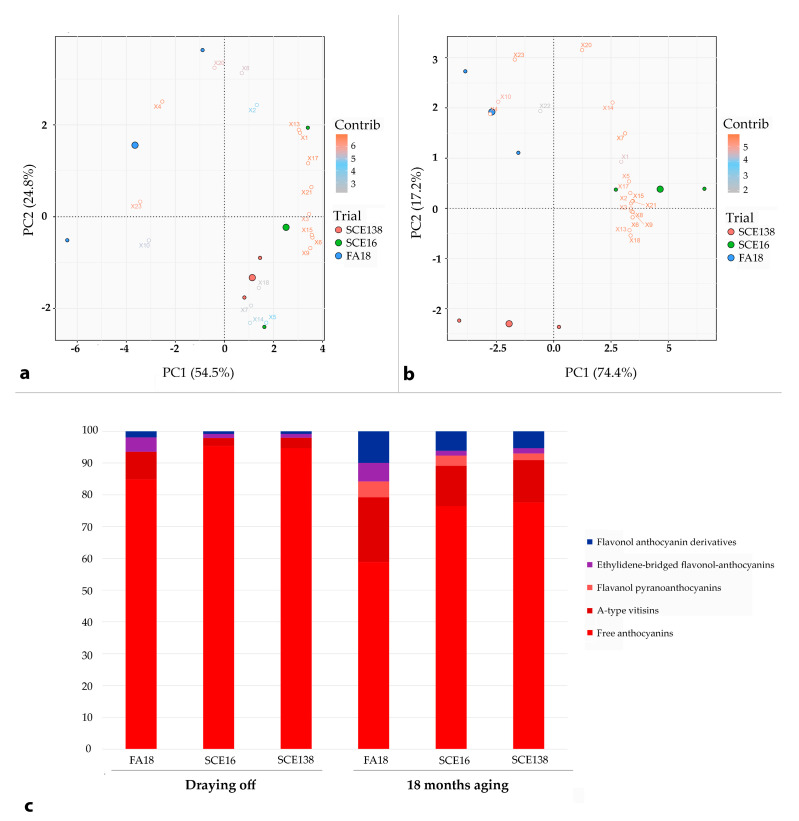
PCA—Negramaro. Principal component diagram of anthocynin-derived red pigments in Negramaro wines SCE138 (green point), SCE 16 (blue point), FA18 (red point), and distribution (percentage) calculated (**a**) at draining off and (**b**) after 18 months of bottle aging; in (**c**), we report the percentage of each pigment as measured by HLPC assays both at draining off and after 18 months of bottle aging. Variables correspond to peaks reported in Table 2.

**Figure 3:**
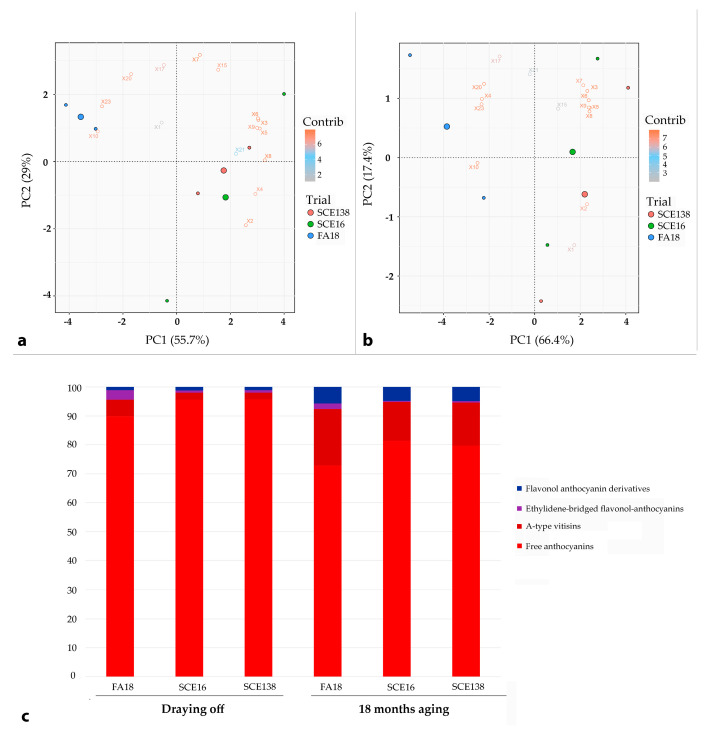
PCA—Aleatico. Principal component diagram of anthocynin-derived red pigments in Aleatico wines SCE138 (green point), SCE 16 (blue point), FA18 (red point), and distribution (percentage) calculated (**a**) at draining off and (**b**) after 18 months of bottle aging; in (**c**), we report the percentage of each pigment as measured by HLPC assays both at draining off and after 18 months of bottle aging. Variables correspond to peaks reported in Table 2.

In order to investigate the influence of the fermentation type on the formation and evolution of anthocyanin derived pigments, involved in the color intensity and stability, PCA analyses were performed on Primitivo, Negramaro, and Aleatico wines at draining off and after 18 months of bottle storage. Moreover, the percentage content of the five different classes of pigments were compared among the wines at the two time-points of aging (Figure 1, Figure 2 and Figure 3). Overall, the mixed fermentation protocol provoked the increasing synthesis of stable pigments in the wines during the vinification process. Indeed, at draining off, the FA18 samples appeared richer in pyranoanthocyanins and ethylidene-bridged compounds, whose content was also enhanced during the bottle aging, thus contributing to the intensity and stability of the color. This was in agreement with the effect of sequential inoculum (delay of 5 days) with *S. bacillaris* CZ1 in the production of wines with a higher level of A-type vitisins [39]. Regarding Primitivo at draining off (Figure 1a), FA18 was characterized by a higher content of vitisin A (10), but also reddish/violet ethylidene-bridged compounds (11, 12, and 19) and bluish pigment (4). On the contrary, SCE16 (and less SCE138) showed greater amounts of free anthocyanins, especially the compounds 6, 9, 17, 20, and 21 together with pyruvic and vinyl derivatives (7 and 16, respectively). Having λ_max_ > 530 nm [31], the relative predominance of the compounds 4, 10, 11, 12, and 19 could partially explain the slightly higher CI in FA18 than SCE wines (Table 1).

Moreover, Negramaro FA18 wines at draining off (Figure 2a,c) were distinguished for having a higher content of stable pigments 4, 10, and 23, which positively affected their color intensity (Table 1). Whereas, SCE wines were separated on the sore plot since more correlated to the free anthocyanins 3, 5, 6, 9, 14, 15, 17, and 21 showing greater factor loadings (>|0.9|) on PC1 and PC2 (Figure 2a).

Finally, with regards to Aleatico, even though the use of *S. bacillaris* in winemaking partially enhanced the formation of stable conjugated forms (especially vitisin A 10 and compound 23) in wines at draining off (Figure 3a,c), this was not enough to intensify and stabilize the color. Indeed, there was no significant variation of CI, H, and CEI among the three wines (Table 1). Furthermore, SCE 18 month-old wines were less clearly separated from FA18 and their relative percentage of pigment families was very close (Figure 3b).

These findings, coupled with the highest H and CEI values in the aged samples (Table 1), indicated a similar and faster color change from red to orange tone and color loss [4]. A possible explanation for this behavior can be attributed to the very low content of anthocyanins (TA) and polyphenols (TP) extracted from grapes in Aleatico wines during both fermentation types.

## 3. Discussion

Wine is the result of a complex biochemical process, that starts with grape harvesting, continues with the alcoholic and malolactic fermentations, wine aging, and bottling [40]. In this process, the diversity and composition of the yeast micro-population may significantly contribute to the organoleptic characteristics of wine, and consequently, those known as terroir. Indeed, modern oenology is increasingly oriented today to the development of technologies and strategies that allow enhancing the typicity and the quality of autochthonous vines. In this regard, one of the most promising ways is the identification of yeasts which are used as a starter in innovative winemaking processes and allow improving the quality of wines. A combination of *S. bacillaris* and *S. cerevisiae* in a sequential fermentation has been described promising to satisfy the modern market and consumer preferences due to its peculiar characteristics [18].

In the present paper, we investigated how mixed fermentation combining the use of *S. bacillaris* with *S. cerevisiae* might influence the color and its stability during aging, one of the most important organoleptic characteristics in red wine, on three of the most typical and commercially important wines in the South of Italy, Primitivo, Negramaro, and Aleatico. In our trials, we first demonstrated that no killer effect exists of the *S. bacillaris* strain FA18 against the chosen *S. cerevisiae* strains (SCE16 and SCE138), thus confirming their suitability in mixed fermentation. Moreover, the kinetics of fermentation and chemical analysis demonstrated that the two *S. cerevisiae* strains have a similar fermentation capacity on all the three cultivars, thus confirming their suitability of combination in *S. bacillaris.*

Our results revealed that mixed fermentation influences both basic parameters and chemical compounds (i.e., pyranoanathocyanins) specifically related to the co-pigments formation and color stabilization. *S. bacillaris* has been described to affect the chemical composition of the musts and wines by producing various metabolites of enological interest [18].

Among these effects, the reduction of ethanol levels in wines has been described when *S. bacillaris* was used coupling with plus *S. cerevisiae* [16,18,25,26]. However, we did not find any variation in the alcoholic degree (% *v*/*v*) (Table 1). Indeed, no significant differences in the ethanol production have been described between mono-*Saccharomyces* and mixed fermentations with some *S. bacillaris* strains. On the contrary, the significant reduction in ethanol is shown when *S. cerevisiae* is added 48 to 72 h after the *S. bacillaris* inoculation, and oxygen is applied during the fermentation process in order to favor the respiration rather than fermentation [16]. Furthermore, the reduction in ethanol for the sequential fermentation is emphasized when the fermentation occurs in a synthetic must medium rather than the natural grape must [41].

Moreover, our data revealed that the fermentation type significantly affected (*p* < 0.05) MA, TA, and TP in the analyzed wines. Pure fermentations allowed a better extraction of anthocyanins and polyphenols as demonstrated by the significant higher value of MA, TA, and TP in SCE16 and SCE138 than in the FA18 samples, especially for Negramaro and Aleatico (Table 1). Despite the aforementioned non-variation of ethanol in our samples, it is known that mixed fermentation of *S. bacillaris* and *S. cerevisiae* leads to a slower development of ethanol in the early stages of winemaking [25,26], thus reducing the extraction of phenolic compounds during the skin maceration [42]. This could partially explain the reduction in phenolic compounds we observed in FA18. Moreover, we detected an even more evident decrease in MA and TA, as well as in TP during aging which in fact is due to the precipitation and degradation phenomena (both oxidative and reductive), that can involve the less stable and oxidizable forms of red wine (such as cyanidin-3-*O*-glucoside and peonidin-3-*O*-glucoside) already described in literature data [7,43].

Most relevant, substantial differences emerged among our wines considering several compounds playing a critical role in the wine color. Indeed, the evolution of wine color is influenced by a number of factors, such as the amount of tannin and acids, grape variety, alcohol and acetaldehyde concentrations, as well as the winemaking and storage conditions of wine [42,44,45]. In particular, the color changes during wine maturation are usually attributed to anthocyanin polymerization reactions and the evolution of co-pigments resulting from interactions between anthocyanins and other compounds at the fermentation phase and during aging [34,35].

Overall, our data highlighted that a 48 h sequential fermentation employing the FA18 *S. bacillaris* in Primitivo, Negramaro, and Aleatico enhances the synthesis of stable anthocyanin pigments, in particular, A-type vitisins and ethylidene-bridge flavonol-anthocyanin adducts, as well as their preservation after 18 months of aging in the bottle. The acidogenic nature of *S. bacillaris*, leading to a more consistent production of pyruvic and acetaldehyde during fermentation, would be responsible for the preferential synthesis of these compounds [25,46]. It is worth pointing out that Primitivo, Negramaro, and Aleatico grapes, used in winemaking, derived from minimal or no canopy management grown vineyards and, thus, were poorer in anthocyanins and polyphenols with respect to conventional conditions, as previously reported in literature [47]. This could motivate the lack of various pigments, such as pinotins, anthocyanin dimers, and trimers, as well as more different vinyl-linked and ethylidene-bridged compounds, compared to wines analyzed by direct injection [33] or after fractionation [7].

Notably, the pyranic structure of malvidin-3-*O*-glucoside pyruvate (10) is recognized as more resistant to the bleaching effect due to SO_2_ than malvidinic free anthocyanins, thereby its presence in wine implies a greater red color stabilization [48]. Furthermore, this vitisin A is resistant to a pH increase [48] and oxidative degradation [49], as well as temperature changes [50]. It is worth noting that, although free anthocyanins more strongly decreased in FA18, mixed fermentation seemed to protect the wine from further non-oxidative degradation reactions. It was confirmed by the relative unstable ethyl linked anthocyanins (11, 12, and 19), whose percentage slightly increased during aging (Figure 1b,c), and the reduced color loss, as proved by the significant lower values of H and CEI than those found in SCE16 and SCE138 after 18 months in the bottle (Table 1). This would be a very important finding from a technological standpoint, since the use of *S. bacillaris* in tandem with *S. cerevisiae* could contribute to mitigate the often-reported rapid change of Primitivo color into orange hue compared to other international wines [7]. In addition, the significant lower values of H and CEI highlighted that Negramaro derived from the mixed inoculum of *S. bacillaris/S. cerevisiae* remained more stable in the color after bottle storage than SCEs (Table 1). This was corroborated by the most pronounced increase in vitisins, ethylidene-bridged pigments, and flavanol-anthocyanin adducts percentage in FA18 aged wines (Figure 2b,c). However, the remarked difference in the color stability of Negramaro wines was less evident respect to Primitivo ones (Table 1), maybe due to the different ethyl linked compounds prevailing in the former (i.e., malvidin-3-*O*-glucoside-8-ethyl-epicatechin) despite the latter samples (i.e., malvidin-3-*O*-glucoside-8-ethyl-epicatechin isomers and peonidin-3-*O*-pcoumaroyl-glucoside-8-ethyl-epicatechin), as well as their relative concentrations (Figure 2).

Notably, at our knowledge, this is the first evidence that mixed fermentation induced the production of ethylidene-bridge flavonol-anthocyanin adducts. Indeed, these adducts have been previously found unstable and intermediate products formed during winemaking and aging, also using different vinification procedures [51,52] or present only at a low concentration, in addition to their importance has been hypothesized [53]. As a matter of fact, these ethylidene linked pigments are associated to a color increase with a shift towards violet [54,55]. Moreover, these pigments undergo further polymerization phenomena, thus leading to an important reduction in astringency [37] that improve the organoleptic quality of the red wines.

## 4. Materials and Methods

### 4.1. Yeast Strains

Two *S. cerevisiae* strains and one *S. bacillaris* strain available at the I.U.V.V.—Institut Universitaire de la Vigne et du Vin Jules Guyot of Dijon (France) were inoculated in red vinification experiments. The two *S. cerevisiae* strains were isolated from ‘Savigninin Jura’ and were coded SCE16 and SCE138, while the *S. bacillaris* strain was isolated from ‘Pinot noir’ in Burgundy and identified as FA18. These strains were previously isolated in Burgundy, characterized, and then selected based on their oenological performances [24,56]. The 5.8S ITS rDNA sequencing confirmed the pure culture condition of these strains and the correct identity of these species [57].

### 4.2. Grape Varieties and Vineyard Conduction

The experiments were carried out in 2017 on three important Apulian *Vitis vinifera* L. red grape varieties: Primitivo, Negramaro, and Aleatico, chosen as used for the most important enological production in Apulia Region, Southern Italy. They were cultivated in an experimental vineyard of the CREA-VE, located in the area around Rutigliano (Bari), Apulia Region, Southern Italy. The vineyards are composed of 13-year-old vines, grafted34 E.M., trained on Gobelet Alberello, and pruned with four spurs of two buds. Plants are planted 1.5 m between rows and 1.0 m in the row. All the vines were cultivated without water supply, chemical inputs, and canopy management. Samples of 130 kg per each variety were hand-harvested at the same time in mid-October, at technical maturity [58]. At harvest, the total soluble solids (TSS) content, A, and pH were as follows: Aleatico: TSS 19.8 °Brix, A 5.9 g/L, pH 3.40; Negramaro: TSS 21 °Brix, A 6.9 g/L, pH 3.38; Primitivo: TSS 25 °Brix, A 7.1 g/L, pH 3.42. The grapes were hand-picked in small pierced plastic crates and immediately crushed and destemmed. After crushing and destemming, 4 g/hL of potassium metabisulphite (the equivalent of 20 mg/L of SO_2_) was added in the unpasteurized must. Organic and inorganic nitrogen sources were added, as described in the laboratory scale protocol of Nisiotou et al. [59]. The obtained must were directly processed for winemaking.

### 4.3. Interaction between Saccharomyces Yeast Strains

In order to test the killer action between the three yeast strains we performed two experiments.

Experiment 1: Cellophane agar layer technique [60]. Sterilized disc of 90 mm diameter of cellophane was laid aseptically over the solidified Yeast Peptone Dextrose Agar (YPDA) medium in culture plates. The plates were laid overnight to allow the excess moisture to evaporate. In addition, 10 µL (at the concentrations of 1.0 × 10^7^ CFU/mL) of each yeast strain (*S. cerevisiae* SCE16, *S. cerevisiae* SCE138, and *S. bacillaris* FA18) were uniformly distributed on the cellophane disc. For each yeast, six plates were produced. Moreover, 10 µL of sterilized YPDA without yeast were used as a control on nine different plates. After 48 h of incubation at 25 °C, the cellophane disc with and without yeast was removed from the plates. On the first three plates previously covered with the cellophane disc with the *S. cerevisiae* strain SCE16, 10 µL (at the concentrations of 1.0 × 10^7^ CFU/mL) of the *S. cerevisiae* strain SCE138 were uniformly distributed and on the other three plates, 10 µL (at the concentrations of 1.0 × 10^7^ CFU/mL) of the *S. bacillaris* strain FA18. The same procedure was used for the six plates covered with the cellophane agar with the saccharomyces strain SCE138 and for the six plates covered with the cellophane disc with the *S. bacillaris* strain FA18. On the plates used as a control, the three yeast strains were uniformly distributed. After incubation at 25 °C for 48 h, the growth of each yeast strain was recorded.

Experiment 2: Growth in liquid media. Each yeast strain was grown in a tube containing liquid YPD for 24 h at 25 °C. Cells were removed by a double centrifugation at 7240 g for 5 min and the supernatant was filtered through a syringe filter (0.22 µm pore size). In addition, 7.5 mL of the filtered supernatant of the *S. cerevisiae* strain SCE138 were placed in two sterilized tubes and added with 7.5 mL of liquid YPD containing the *S. cerevisiae* strain SCE16 or liquid YPD containing the *S. bacillaris* strain FA18, both at the concentration of 1.0 × 10^6^ CFU/mL. The same procedure was followed using the filtered supernatant of SCE16 and FA18 added with liquid YPD containing living cells of other yeast strains. The tubes were placed on an orbital shaker at 25 °C for 48 h. After 18, 24, 42, and 48 h, two aliquots of 1 mL each were aseptically withdrawn from each tube. Using a spectrophotometer (Thermo Scientific NanoDrop2000) the growth of yeast cultures was monitored by measuring the optical density (OD) at 600 nm. For each aliquot, five replicates/lectures have been performed and the average values were used to plot the growth curve of each yeast strain in the presence of the filtered supernatant of another yeast strain.

### 4.4. Pilot Scale Fermentation Procedure

Pilot scale vinification trials of 20 kg (equal solid/liquid ratio in each trial) were conducted in stainless steel fermenters. The must obtained, corresponding to 18 Lt from each sample of single variety were fermented separately following a standard red winemaking procedure and three independent replicates for each trial were finally carried out.

Each trial was as follows: (i) Mono-SCE16 inoculation (SCE16), (ii) mono-SCE138 inoculation (SCE138), and (iii) a mixed fermentation where the two *S. cerevisiae* (SCE16 and SCE138, 1:1) were added together 48 h after the inoculation of *S. bacillaris* (FA18). Each yeast strain was inoculated at a starting concentration of about 5 × 10^6^ CFU/mL. The possible lack of nutrients was avoided through a standard addition of nitrogen nutrients and enzymatic cofactors into the fermenting juice (20 g/hL of organic nitrogen). This was applied when sugar consumption reached 50 gr/Lt in the mono-SCE16 and -SCE138, while it was implemented in the mixed fermentations (FA18) after 48 h, when the two *S. cerevisiae* strains were inoculated, thus to enhance the *Saccharomyces* metabolic activities, avoiding nutrients depletion and preventing *Saccharomyces* growth arrest.

The fermentation proceeded at a constant temperature of 25 ± 0.5 °C, performing manual pushing down of the pomace cap three times a day during the first half of the fermentation and two times a day until the end. Fermentation kinetics were measured, checking the level of sugar consumption (°Babo), utilizing a standard hydrometer. Macerations and fermentations were considered ended when residual sugar levels, measured with a hydrometer (Babo Klosterneuburg Mostimeter), reached 0 °Babo (8–10 days). The complete fermented must was pressed (up to 2–3 bar) and kept in the cellar for 2 days before storage in a refrigerated room (4–5 °C) to allow the residual solid parts (solid lees) to settle down. The wines were racked after a week to remove the solid lees. Consequently, wines were poured in 0.75 L glass bottles, supplemented with potassium metabisulphite to achieve a final concentration of 80 mg/L of total SO_2_. The wines were stored at a constant temperature of 15 °C and analyzed at draining off and after 18 months of aging to assess the color stabilization and the variation of chromatic characteristics.

### 4.5. Chemical Analysis

A chemical analysis on wine and must was performed according to the EEC regulation 2676/90, as reported by the International Organization for Vine and Wine (OIV, 2018: https://www.oiv.int/en/technical-standards-and-documents/methods-of-analysis/compendium-of-international-methods-of-analysis-of-wines-and-musts-2-vol (accessed on 24 April 2020)). Titratable acidity, A (g/L of tartaric acid) was measured following OIV MA-AS313-01 R2015 par.5.3;pH: OIV MA-AS313-15 R2011; volatile acidity, VA (g/L of acetic acid): OIV MA-AS313-02 R2015; alcoholic degree, ET (% *v*/*v*): OIV MA-AS312-01A R2016 par. 4C. The wine color was assessed by the Glories chromatic parameters [61]: Color intensity (CI) was calculated as the sum of absorbance (λ_420_ + λ_520_ + λ_620_ nm); hue (H) was defined as the ratio λ_420_/λ_520_ nm, while the color evolution index (CEI) was calculated as (λ_420_−λ_520_ nm)/λ_420_ nm.

### 4.6. Phenolic Indexes

Total polyphenols (TP), total anthocyanins (TA), and monomeric anthocyanins (MA) were measured spectrophotometrically to assess the phenolic wine composition and the overall chromatic characteristics.

TP was determined following the method suggested by Waterhouse et al. [62]. From each sample, 20 µL were collected in separate cuvettes, and mixed with 1.58 mL water and 100 µL of Folin-Ciocalteu reagent. After 5 min, 300 µL Na2CO3 10% were added and the solution was shacked. The absorbance of each solution was read at λ_750_ nm against a blank after waiting for 2 h at 20 °C. A calibration curve (R2 = 0.9264) was set with a polyphenolic concentration between 0–3000 mg/L of gallic acid, considering the effective range of the assay. Results were reported as mg/L of gallic acid equivalents (GAE).

TA was determined as already reported [38]. Briefly, the samples were diluted in a solution consisting of 70/30/1 (*v*/*v*/*v*) ethanol/water/HCl. The relative absorbance for each sample was measured at λ_max_ of 540 nm. The total anthocyanin content was expressed as mg/L of malvidin-3-*O*-glucoside equivalents.

Finally, MA was measured by the spectrophotometric determination reported by Lee et al. [63]. Briefly, all the dilutions were performed in 50 mL volumetric flasks. At the beginning, the appropriate dilution factor by diluting the test portion with a pH 1.0 buffer was determined until absorbance at λ_max_ of 520 nm was within the linear range (between 0.2 and 1.4 AU). Using the appropriate dilution factor, two dilutions of each test sample, either for pH 1.0 (potassium chloride, 0.0025 M) or pH 4.5 (sodium acetate, 0.4 M) buffers were prepared. Hence, the determination proceeded through pH 1.0 and 4.5 buffer dilutions of the samples, reading them both at λ_max_ of 520 and 700 nm. The measure at 700 nm was considered a wine haze correction of the reading at 520 nm. The content of anthocyanin pigments was expressed as mg/L of cyanidin-3-*O*-gluoside equivalents.

### 4.7. Anthocyanin Profile Determined by HPLC-DAD-MS

An HPLC 1100 equipped with a DAD and XCT-trap Plus mass detector (Agilent Technologies, Palo Alto, CA., USA) coupled with an ESI interface was used. The reversed stationary phase employed was a Zorbax C18 5 μm (250 × 4.6 mm i.d., Agilent Technologies) with a pre-column Gemini C18 5 μm (4 × 2 mm i.d., Phenomenex, Castel Maggiore, Bologna, Italy). The following gradient system was used with water/formic acid (90:10, *v*/*v*) (solvent A) and acetonitrile (solvent B): 0 min, 95% A— 5% B; 10 min, 87% A —13% B; 20 min, 85% A—15% B; 30 min, 78% A—22% B; 50 min 78% A—22% B; 55 min 5% A— 95% B; stop time at 70 min. Finally, the column was re-equilibrated with the initial solvent mixture for 15 min. The flow was maintained at 0.7 mL/min; the sample injection was 5 μL. Wine samples were filtered (0.2 μm RC syringe filters, Phenomenex) before the HPLC analysis. The diode array detection was between 250 and 650 nm, and absorbance was recorded at 520 nm. The positive electrospray mode was used for ionization of the molecules with capillary voltage at 4000 V and skimmer voltage at 30 V. The nebulizer pressure was 40 psi and the nitrogen flow rate was 9 L/min. The temperature of drying gas was 350 °C. The monitored mass range was from *m*/*z* 100 to 1200.

Free and co-pigmented anthocyanins were identified by matching the chromatographic elution order, molecular ions, and MS/MS fragments with those reported in the literature [7]. Semi-quantitation was performed using extracted ion chromatograms (EIC): For each compound, the EIC at the corresponding molecular ion was obtained and the relevant peak was integrated (Supplementary Table S2). Subsequently, peak areas were summed with respect to the type of pigment to calculate the percentage content of the different classes determined in the wines.

### 4.8. Organic Acids Determination by HPLC-UV

An HPLC 1100 equipped with a VWD detector (Agilent Technologies, Palo Alto, CA, USA) was used. The reversed stationary phase employed was a Synergy Hydro-RP-80A 5 μm (250 × 4.6 mm i.d., Phenomenex, Castel Maggiore, Bologna, Italy) with a pre-column Gemini C18 5 μm (4 × 2 mm i.d., Phenomenex, Castel Maggiore, Bologna, Italy). The separation was conducted in an isocratic mode using water/orthophosphoric acid (0.1%) as the mobile phase. The flow was maintained at 0.7 mL/min and sample injection was 5 μL. Wine samples were 2-folds diluted and filtered (0.2 μm RC syringe filters, Phenomenex) before the HPLC analysis. Absorbance was recorded at 210 nm.

### 4.9. Statistical Analysis

Data were analyzed using the R package software (version 3.4.0). Specifically, after testing their normal distribution by the Mardia test, a two-way multivariate analysis of variance (MANOVA) was performed on the chemical composition data in order to evaluate the effect of the factors fermentation type and aging, whose significance was discussed in the text. Tukey’s HSD post-hoc test was used to separate the means (*p* < 0.05) when the interaction between the factors was significant (Table 1). Furthermore, the principal component analysis (PCA) of the dataset was performed on semi-quantified HPLC-anthocyanin profiles of each wine at draining off and after 18 months in the bottle to explore qualitative differences. In the PCA, only the first two components were considered accounting for more than 80% of the total variance explained.

## 5. Conclusions

In conclusion, the presented results highlighted that the use of *S. bacillaris* in tandem with *S. cerevisiae* has positively contributed to the evolution and stability of the wine color during the aging process. Although preliminary, our data are a further step that highlight the applicative technological potential of mixed fermentations with *S. bacillaris*. [18]. Indeed, our results support the importance of mixed fermentations to enhance the organoleptic characteristics (such as color intensity and stability) and shelf-life of wines that belong to the winemaking tradition. In particular, mixed fermentation with *S. bacillaris* might represent a valuable technological tool for mitigating the often reported rapid change of the color of some mono-varietal wines (such as Primitivo) towards an orange-brown hue. Moreover, we highlighted new clues on the impact of individual components produced in the presence of different starters on the final wine quality. This is a small pilot scale fermentation trial, but as a future perspective, the possibility of testing mixed cultures on different musts while also studying more in-depth yeast interactions, offer the opportunity to evaluate their benefits and limitations in order to select the best starters capable of fully enhancing the qualities of the resulting wines.

## Figures and Tables

**Table 1:** Chemical analysis and polyphenolic indexes of Primitivo, Negramaro, and Aleatico at draining off (A) and after 18 months of bottle aging (B).

	Primitivo A			Primitivo B		
	SCE16	SCE138	FA18	SCE16	SCE138	FA18
CI	1.19 ± 0.10 ^#^	1.04 ± 0.15	1.24 ± 0.05	0.92 ± 0.07	0.80 ± 0.12	0.96 ± 0.04
MA (mg/L)	176 ± 5ac	157 ± 11ab	148 ± 6b	121 ± 3c	109 ± 3d	102 ± 10d
TA (mg/L)	324 ± 16a	292 ± 20ab	294 ± 11ab	182 ± 17b	158 ± 16c	165 ± 12bc
TP (mg/L)	2390 ± 70a	2230 ± 60ab	2430 ± 80a	2081 ± 40b	1900 ± 100c	2110 ± 50b
pH	3.13 ± 0.03	3.21 ± 0.04	3.26 ± 0.05	3.39 ± 0.03	3.36 ± 0.04	3.39 ± 0.02
A (g/L)	7.81 ± 0.10	7.62 ± 0.15	7.67 ± 0.08	7.7 ± 0.2	7.50 ± 0.07	7.55 ± 0.07
ET (% *v*/*v*)	15.52 ± 0.12	14.9 ± 0.5	15.21 ± 0.12	15.31 ± 0.10	14.7 ± 0.4	15.0 ± 0.2
VA (g/L)	0.29 ± 0.04ab	0.25 ± 0.02c	0.28 ± 0.02b	0.23 ± 0.03c	0.24 ± 0.03c	0.32 ± 0.02a
H	0.283 ± 0.019c	0.24 ± 0.04d	0.256 ± 0.015cd	0.597 ± 0.003a	0.606 ± 0.006a	0.562 ± 0.009b
CEI	−2.6 ± 0.2c	−3.2 ± 0.7c	−2.99 ± 0.17c	−0.675 ± 0.008a	−0.650 ± 0.017a	−0.76 ± 0.02b
	Negramaro A			Negramaro B		
	**SCE16**	**SCE138**	**FA18**	**SCE16**	**SCE138**	**FA18**
CI	0.553 ± 0.018b	0.58 ± 0.03b	0.743 ± 0.019a	0.425 ± 0.014c	0.45 ± 0.02c	0.571 ± 0.015b
MA (mg/L)	117 ± 4a	107 ± 5a	98 ± 4b	97 ± 5b	72 ± 10c	69 ± 11c
TA (mg/L)	211 ± 3a	202 ± 2ab	189 ± 7b	120 ± 5c	110 ± 9cd	103 ± 6d
TP (mg/L)	2040 ± 90	1900 ± 70	1790 ± 100	2060 ± 70	1830 ± 90	1600 ± 100
pH	3.40 ± 0.05	3.30 ± 0.03	3.36 ± 0.03	3.56 ± 0.06	3.52 ± 0.04	3.54 ± 0.02
A (g/L)	6.07 ± 0.04b	6.10 ± 0.03ab	6.4 ± 0.03a	5.7 ± 0.03c	6.00 ± 0.02b	6.2 ± 0.03a
ET (% *v*/*v*)	12.3 ± 0.2	12.52 ± 0.12	12.33 ± 0.06	12.3 ± 0.3	12.43 ± 0.06	12.26 ± 0.13
VA (g/L)	0.21 ± 0.02b	0.23 ± 0.03b	0.23 ± 0.04b	0.20 ± 0.02b	0.21 ± 0.02b	0.38 ± 0.03a
H	0.435 ± 0.016d	0.437 ± 0.016d	0.412 ± 0.015c	0.73 ± 0.03a	0.717 ± 0.006a	0.694 ± 0.012b
CEI	−1.30 ± 0.08d	−1.29 ± 0.08d	−1.13 ± 0.06c	−0.37 ± 0.06b	−0.395 ± 0.012ab	−0.41 ± 0.03b
	Aleatico A			Aleatico B		
	**SCE16**	**SCE138**	**FA18**	**SCE16**	**SCE138**	**FA18**
CI	0.43 ± 0.05	0.40 ± 0.08	0.49 ± 0.03	0.33 ± 0.04	0.31 ± 0.06	0.39 ± 0.02
MA (mg/L)	95 ± 6a	97 ± 3a	85 ± 3b	70 ± 5bc	80 ± 7b	65 ± 4c
TA (mg/L)	157 ± 8a	165 ± 8a	152 ± 6ab	97 ± 9cb	103 ± 7b	92 ± 4c
TP (mg/L)	1700 ± 50a	1720 ± 20a	1600 ± 60b	1430 ± 50bc	1440 ± 80bc	1320 ± 40c
pH	3.26 ± 0.04	3.27 ± 0.06	3.22 ± 0.04	3.42 ± 0.02	3.43 ± 0.02	3.38 ± 0.04
A (g/L)	5.61 ± 0.02b	5.59 ± 0.10b	6.18 ± 0.04a	5.50 ± 0.14b	5.50 ± 0.02b	6.05 ± 0.07a
ET (% *v*/*v*)	12.0 ± 0.18	11.9 ± 0.03	11.7 ± 0.06	11.95 ± 0.13	11.83 ± 0.04	11.60 ± 0.02
VA (g/L)	0.22 ± 0.02b	0.21 ± 0.01b	0.26 ± 0.04a	0.17 ± 0.01c	0.18 ± 0.01c	0.24 ± 0.01ab
H	0.543 ± 0.017	0.527 ± 0.019	0.564 ± 0.016	0.760 ± 0.013	0.753 ± 0.018	0.78 ± 0.03
CEI	−0.84 ± 0.03	−0.90 ± 0.03	−0.806 ± 0.013	−0.22 ± 0.03	−0.23 ± 0.04	−0.26 ± 0.05

Each value was calculated as means of three independent replicates ± *^#^*standard deviation at *p* < 0.05. Different letters on the same line are significantly different at a 5% level (Tukey’s HSD post-hoc test). CI: Color intensity; MA: Monomeric anthocyanins; TA: Total anthocyanins; TP: Total polyphenols; A: Total acidity; ET: Alcoholic degree; VA: Volatile acidity; H: Hue; CEI: Color evolution index.

**Table 2:** Chromatographic and mass spectral data of the identified anthocyanin compounds.

Peak	RT	Compound	[M]^+^ (*m*/*z*)	MS/MS
1	9.896	(epi)-catechin-peonidin-3-*O*-glucoside	751	589, 463, 437
2	10.603	(epi)-catechin-malvidin-3-*O*-glucoside	781	619, 493, 467
3	11.356	delphinidin-3-*O*-glucoside	465	303
4	12.236	di(epi)catechin-malvidin-3-*O*-glucoside	1069	907, 781, 619
5	13.511	cyanidin-3-*O*-glucoside	449	287
6	14.975	petunidin-3-*O*-glucoside	476	317
7	16.458	petunidin-3-*O*-glucoside pyruvate	547	385
8	17.520	peonidin-3-*O*-glucoside	463	301
9	19.147	malvidin-3-*O*-glucoside	493	331
10	22.096	malvidin-3-*O*-glucoside pyruvate	561	399
11	28.755	malvidin-3-*O*-glucoside-8-ethyl-(epi)catechin	809	647,519,357
12	30.251	malvidin-3-*O*-glucoside-8-ethyl-(epi)catechin	809	647,519,357
13	31.197	peonidin-3-*O*-(*p*-coumaryl)-glucoside pyruvate	677	369
14	31.957	peonidin-3-*O*-acetylglucoside	505	301
14	31.957	malvidin-3-*O*-(*p*-coumaryl)-glucoside pyruvate	707	399
15	33.237	malvidin-3-*O*-acetylglucoside	535	331
16	34.843	malvidin-3-*O*-acetylglucoside-4-vinyl-(epi)catechin	847	643,491
17	35.622	malvidin-3-*O*-caffeoylglucoside	655	331
17	35.622	cyanidin-3-*O*-(*p*-coumaryl)-glucoside	595	287
18	36.914	petunidin-3-*O*-(*p*-coumaryl)-glucoside	625	317
18	36.914	malvidin-3-*O*-*cis*-(*p*-coumaryl)-glucoside	639	331
19	37.694	peonidin-3-*O*-(*p*-coumaryl)-glucoside-8-ethyl-(epi)catechin	925	635,617,327
20	39.841	peonidin-3-*O*-*trans*-(*p*-coumaryl)-glucoside	609	301
21	41.231	malvidin-3-*O*-*trans*-(*p*-coumaryl)-glucoside	639	331
22	42.355	malvidin-3-*O*-glucoside-4-vinyl-(epi)catechin	805	643,491
23	49.702	malvidin-3-*O*-(*p*-coumaroyl)-glucoside-8-ethyl-(epi)catechin	955	665,647,357

## Data Availability

The data presented in this study are available on request from the corresponding author.

## Supplementary Materials

The following are available online. Table S1: Concentration of organic acids in the studied wines; Table S2: Quantities of the identified anthocyanins into wines; Figure S1: Cell concentration of each yeast strain grown in liquid YPD amended with filtered supernatant obtained from the growth of each yeast strain on YPD. Data are the mean value of two replicates and five lectures/replicates. The time in hours is reported on abscises, while the ordinate axes reported the 10 logarithms of the number of cells per mL; Figure S2: Fermentation kinetics of Primitivo, Negroamaro, and Aleatico in pilot scale conditions: The days of fermentation are reported on abscises, while °Babo is reported on the ordinates. SCE16 and SCE138: Mono *S. cerevisiae* fermentations; FA18: Mixed fermentation of *S. bacillaris* FA18 and the two *S. cerevisiae* strains (48 h of delay). Red arrows indicate the addition of the two *S.cerevisiae* strains.

## Sample Availability

Samples are available from the authors.

## References

[1] Capozzi V., Garofalo C., Chiriatti M.A., Grieco F., Spano G. (2015). Microbial terroir and food innovation: The case of yeast biodiversity in wine. Microbiol. Res..

[2] Hernández B., Sáenz C., Alberdi C., Alfonso S., Diñeiro J.M. (2016). Colour Evolution of Rosé Wines after Bottling. S. Afr. J. Enol. Vitic..

[3] Salinas M.R., Garijo J., Pardo F., Zalacain A., Alonso G.L. (2003). Color, polyphenol, and aroma compounds in rosé wines after prefermentative maceration and enzymatic treatments. Am. J. Enol. Vitic..

[4] Benucci I., Cerreti M., Liburdi K., Nardi T., Vagnoli P., Ortiz-Julien A., Esti M. (2018). Pre-fermentative cold maceration in presence of non- Saccharomyces strains: Evolution of chromatic characteristics of Sangiovese red wine elaborated by sequential inoculation. Food Res. Int..

[5] Canals R., Llaudy M.C., Valls J., Canals A.J.M., Zamora F. (2005). Influence of Ethanol Concentration on the Extraction of Color and Phenolic Compounds from the Skin and Seeds of Tempranillo Grapes at Different Stages of Ripening. J. Agric. Food Chem..

[6] Torchio F., Segade S.R., Gerbi V., Cagnasso E., Rolle L. (2011). Changes in chromatic characteristics and phenolic composition during winemaking and shelf-life of two types of red sweet sparkling wines. Food Res. Int..

[7] Dipalmo T., Crupi P., Pati S., Clodoveo M.L., Di Luccia A. (2016). Studying the evolution of anthocyanin-derived pigments in a typical red wine of Southern Italy to assess its resistance to aging. LWT.

[8] Pérez-Magariño S., González-Sanjosé M.L. (2004). Evolution of Flavanols, Anthocyanins, and Their Derivatives during the Aging of Red Wines Elaborated from Grapes Harvested at Different Stages of Ripening. J. Agric. Food Chem..

[9] Jackson D.I., Lombard B.P. (1993). Environmental and management practices affecting grape composition and wine quality: A review. Am. J. Enol. Vitic..

[10] Pérez-Lamela C., García-Falcón M., Simal-Gandara J., Orriols-Fernández I. (2007). Influence of grape variety, vine system and enological treatments on the colour stability of young red wines. Food Chem..

[11] Asenstorfer R.E., Markides A.J., Iland P.G., Jones G.P. (2003). Formation of vitisin A during red wine fermentation and maturation. Aust. J. Grape Wine Res..

[12] Jolly N., Augustyn O., Pretorius I. (2017). The Role and Use of Non-Saccharomyces Yeasts in Wine Production. S. Afr. J. Enol. Vitic..

[13] Loira I., Morata A., Comuzzo P., Callejo M.J., González C., Calderón F., Suárez-Lepe J.A. (2015). Use of Schizosaccharomyces pombe and Torulaspora delbrueckii strains in mixed and sequential fermentations to improve red wine sensory quality. Food Res. Int..

[14] Ortega A.F., Lopez-Toledano A., Mayen M., Merida J., Medina M. (2003). Changes in Color and Phenolic Compounds during Oxidative Aging of Sherry White Wine. J. Food Sci..

[15] Duarte F., Pimentel N.H., Teixeira A., Fonseca A. (2012). Saccharomyces bacillaris is not a synonym of Candida stellata: Reinstatement as Starmerella bacillaris comb. nov. Antonie van Leeuwenhoek.

[16] Englezos V., Rantsiou K., Cravero F., Torchio F., Ortiz-Julien A., Gerbi V., Rolle L., Cocolin L. (2016). Starmerella bacillaris and Saccharomyces cerevisiae mixed fermentations to reduce ethanol content in wine. Appl. Microbiol. Biotechnol..

[17] Junior W.J.F.L., Nadai C., Crepalde L.T., De Oliveira V.S., De Matos A.D., Giacomini A., Corich V. (2019). Potential use of Starmerella bacillaris as fermentation starter for the production of low-alcohol beverages obtained from unripe grapes. Int. J. Food Microbiol..

[18] Englezos V., Giacosa S., Rantsiou K., Rolle L., Cocolin L. (2017). Starmerella bacillaris in winemaking: Opportunities and risks. Curr. Opin. Food Sci..

[19] Sipiczki M. (2003). Candida zemplinina sp. nov., an osmotolerant and psychrotolerant yeast that ferments sweet botrytized wines. Int. J. Syst. Evol. Microbiol..

[20] Torija M.-J., Rozès N., Poblet M., Guillamón J.M., Mas A. (2001). Yeast population dynamics in spontaneous fermentations: Comparison between two different wine-producing areas over a period of three years. Antonie van Leeuwenhoek.

[21] Romancino D.P., Di Maio S., Muriella R., Oliva D. (2008). Analysis of non-Saccharomycesyeast populations isolated from grape musts from Sicily (Italy). J. Appl. Microbiol..

[22] Fleet G.H. (2008). Wine yeasts for the future. FEMS Yeast Res..

[23] Comitini F., Gobbi M., Domizio P., Romani C., Lencioni L., Mannazzu I., Ciani M. (2011). Selected non-Saccharomyces wine yeasts in controlled multistarter fermentations with Saccharomyces cerevisiae. Food Microbiol..

[24] Sadoudi M., Tourdot-Maréchal R., Rousseaux S., Steyer D., Gallardo-Chacón J.-J., Ballester J., Vichi S., Guérin-Schneider R., Caixach J., Alexandre H. (2012). Yeast–yeast interactions revealed by aromatic profile analysis of Sauvignon Blanc wine fermented by single or co-culture of non-Saccharomyces and Saccharomyces yeasts. Food Microbiol..

[25] Englezos V., Rantsiou K., Cravero F., Torchio F., Giacosa S., Ortiz-Julien A., Gerbi V., Rolle L., Cocolin L. (2018). Volatile profiles and chromatic characteristics of red wines produced with Starmerella bacillaris and Saccharomyces cerevisiae. Food Res. Int..

[26] Englezos V., Cocolin L., Rantsiou K., Ortiz-Julien A., Bloem A., Dequin S., Camarasa C. (2018). Specific Phenotypic Traits ofStarmerella bacillarisRelated to Nitrogen Source Consumption and Central Carbon Metabolite Production during Wine Fermentation. Appl. Environ. Microbiol..

[27] Magyar I., Nyitrai-Sárdy D., Leskó A., Pomázi A., Kállay M. (2014). Anaerobic organic acid metabolism of Candida zemplinina in comparison with Saccharomyces wine yeasts. Int. J. Food Microbiol..

[28] Lleixà J., Manzano M., Mas A., Portillo M.C. (2016). Saccharomyces and non-Saccharomyces Competition during Microvinification under Different Sugar and Nitrogen Conditions. Front. Microbiol..

[29] Bell S.-J., Henschke P.A. (2005). Implications of nitrogen nutrition for grapes, fermentation and wine. Aust. J. Grape Wine Res..

[30] Medina K., Boido E., Dellacassa E., Carrau F. (2012). Growth of non-Saccharomyces yeasts affects nutrient availability for Saccharomyces cerevisiae during wine fermentation. Int. J. Food Microbiol..

[31] Hazelwood L.A., Daran J.-M., Van Maris A.J.A., Pronk J.T., Dickinson J.R. (2008). The Ehrlich Pathway for Fusel Alcohol Production: A Century of Research on Saccharomyces cerevisiae Metabolism. Appl. Environ. Microbiol..

[32] Morata A., Loira I., Heras J.M., Callejo M.J., Tesfaye W., González C., Suárez-Lepe J.A., Barrado A.D.M. (2016). Yeast influence on the formation of stable pigments in red winemaking. Food Chem..

[33] Pati S., Crupi P., Savastano M.L., Benucci I., Esti M. (2020). Evolution of phenolic and volatile compounds during bottle storage of a white wine without added sulfite. J. Sci. Food Agric..

[34] Mateus N., Freitas V. (2001). Evolution and Stability of Anthocyanin-Derived Pigments during Port Wine Aging. J. Agric. Food Chem..

[35] Mateus N., Silva A.M.S., Vercauteren J., Freitas V. (2001). Occurrence of anthocyanin-derived pigments in red wines. J. Agric. Food Chem..

[36] He F., Liang N.-N., Mu L., Pan Q.-H., Wang J., Reeves M.J., Duan C.-Q. (2012). Anthocyanins and Their Variation in Red Wines I. Monomeric Anthocyanins and Their Color Expression. Molecules.

[37] He F., Liang N.-N., Mu L., Pan Q.-H., Wang J., Reeves M.J., Duan C.-Q. (2012). Anthocyanins and Their Variation in Red Wines II. Anthocyanin Derived Pigments and Their Color Evolution. Molecules.

[38] Coletta A., Trani A., Faccia M., Punzi R., Dipalmo T., Crupi P., Antonacci D., Gambacorta G. (2013). Influence of viticultural practices and winemaking technologies on phenolic composition and sensory characteristics of Negroamaro red wines. Int. J. Food Sci. Technol..

[39] Romboli Y., Mangani S., Buscioni G., Granchi L., Vincenzini M. (2015). Effect of Saccharomyces cerevisiae and Candida zemplinina on quercetin, vitisin A and hydroxytyrosol contents in Sangiovese wines. World J. Microbiol. Biotechnol..

[40] Romano P. (2003). Function of yeast species and strains in wine flavour. Int. J. Food Microbiol..

[41] Lemos W.J., Bovo B., Nadai C., Crosato G., Carlot M., Favaron F., Giacomini A., Corich V. (2016). Biocontrol Ability and Action Mechanism of Starmerella bacillaris (Synonym Candida zemplinina) Isolated from Wine Musts against Gray Mold Disease Agent Botrytis cinerea on Grape and Their Effects on Alcoholic Fermentation. Front. Microbiol..

[42] Ribéreau-Gayon P., Dubourdieu D., Donèche B., Lonvaud A. (2017). Traité d’oenologie. Microbiologie du vin Vinifications.

[43] Canuti V., Puccioni S., Giovani G., Salmi M., Rosi I., Bertuccioli M. (2012). ffect of oenotannin addition on the composition of sangiovese wines from grapes with different characteristics. Am. J. Enol. Vitic..

[44] Suárez-Lepe J., Morata A. (2012). New trends in yeast selection for winemaking. Trends Food Sci. Technol..

[45] Božič J.T., Butinar L., Albreht A., Vovk I., Korte D., Vodopivec B.M. (2020). The impact of Saccharomyces and non-Saccharomyces yeasts on wine colour: A laboratory study of vinylphenolic pyranoanthocyanin formation and anthocyanin cell wall adsorption. LWT.

[46] Fulcrand H., Dueñas M., Salas E., Cheynier V. (2006). Phenolic reactions during winemaking and aging. Am. J. Enol. Vitic..

[47] Di Profio F., Reynolds A.G., Kasimos A., Lopes P., Marques J., Lino J., Coelho J., Alves C., Roseira I., Mendes A. (2011). Canopy Management and Enzyme Impacts on Merlot, Cabernet franc, and Cabernet Sauvignon. II. Wine Composition and Quality. Am. J. Enol. Vitic..

[48] Alcalde-Eon C., Escribano-Bailón M.T., Santos-Buelga C., Rivas-Gonzalo J.C. (2006). Changes in the detailed pigment composition of red wine during maturity and ageing. Anal. Chim. Acta.

[49] Bakker J., Timberlake C.F. (1997). Isolation, Identification, and Characterization of New Color-Stable Anthocyanins Occurring in Some Red Wines. J. Agric. Food Chem..

[50] Sarni-Manchado P., Fulcrand H., Souquet J.-M., Cheynier V., Moutounet M. (1996). Stability and Color of Unreported Wine Anthocyanin-derived Pigments. J. Food Sci..

[51] Rivas-Gonzalo J.C., Bravo-Haro S., Santos-Buelga C. (1995). Detection of Compounds Formed through the Reaction of Malvidin 3-Monoglucoside and Catechin in the Presence of Acetaldehyde. J. Agric. Food Chem..

[52] Escribano-Bailon T., Alvarez-Garcia M., Rivas-Gonzalo J., Heredia F.J., Santos-Buelga C. (2001). Color and stability of pig-ments derived from the acetaldehyde-mediated condensation between malvidin 3-O-glucoside and (+)-catechin. J. Agric. Food Chem..

[53] Drinkine J., Lopes P., Kennedy J.A., Teissedre P.-L., Saucier C. (2007). Ethylidene-Bridged Flavan-3-ols in Red Wine and Correlation with Wine Age. J. Agric. Food Chem..

[54] Dallas C., Ricardo-Da-Silva A.J.M., Laureano O. (1996). Products Formed in Model Wine Solutions Involving Anthocyanins, Procyanidin B2, and Acetaldehyde. J. Agric. Food Chem..

[55] Es-Safi N.-E., Le Guernevé C., Cheynier V., Moutounet M. (2000). New phenolic compounds formed by evolution of (+)-catechin and glyoxylic acid in hydroalcoholic solution and their implication in color changes of grape-derived foods. J. Agric. Food Chem..

[56] Gobert A., Tourdot-Maréchal R., Morge C., Sparrow C., Liu Y., Quintanilla-Casas B., Vichi S., Alexandre H. (2017). Non-Saccharomyces Yeasts Nitrogen Source Preferences: Impact on Sequential Fermentation and Wine Volatile Compounds Profile. Front. Microbiol..

[57] Diguta C., Vincent B., Guilloux-Benatier M., Alexandre H., Rousseaux S. (2011). PCR ITS-RFLP: A useful method for identifying filamentous fungi isolates on grapes. Food Microbiol..

[58] Jones G.V., Davis R.E. (2000). Climate influences on grapevine phenology, grape composition, and wine production and quality for Bordeaux, France. Am. J. Enol. Vitic..

[59] Nisiotou A., Sgouros G., Mallouchos A., Nisiotis C.-S., Michaelidis C., Tassou C., Banilas G. (2018). The use of indigenous Saccharomyces cerevisiae and Starmerella bacillaris strains as a tool to create chemical complexity in local wines. Food Res. Int..

[60] Gibbs J.N. (1967). A Study of the Epiphytic Growth Habit of Fomes annosus. Ann. Bot..

[61] Glories Y. (1984). La couleur des vins rouges. 2e partie: Mesure, origine et interprétation. OENO ONE.

[62] Waterhouse A.L. (2002). Determination of Total Phenolics by Folin-Ciocalteau Colorimetry. Current Protocols in Food Analytical Chemistry.

[63] Lee J.M., Durst R.W., Wrolstad R.E. (2006). Determination of total monomeric anthocyanin pigment content of fruit juices, beverages, natural colorants, and wines by the pH differential method: Collaborative study. J. AOAC Int..

