# Supplementary material for: Color Stabilization of Apulian Red Wines through the Sequential Inoculation of Starmerella bacillaris and Saccharomyces cerevisiae
Source: Molecules. 2021 Feb 9;26(4):907. doi: 10.3390/molecules26040907 (PMC7915498; doi:10.3390/molecules26040907)
Supplement: Supplementary file 1 [file molecules-26-00907-s001.pdf]

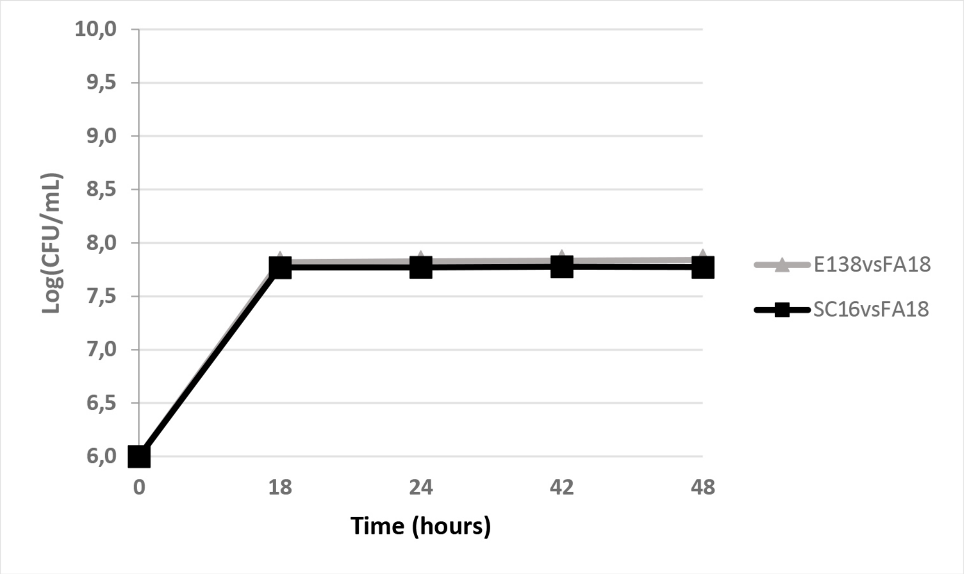

Log(CFU/mL)
10,0
9,5
9,0
8,5
8,0
7,5
7,0
6,5
6,0
0
18
24
42
48
Time (hours)
E138vsFA18
SC16vsFA18


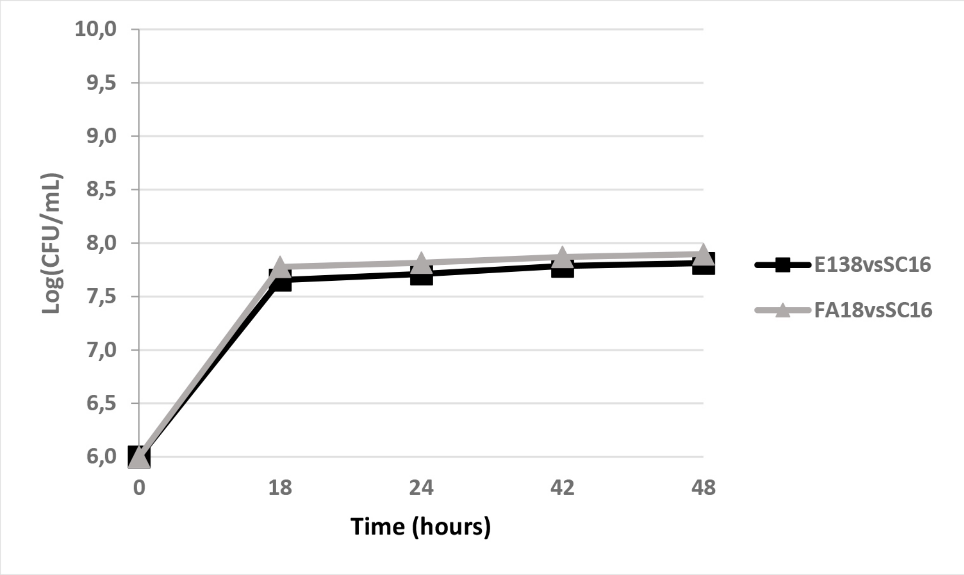

Log(CFU/mL)
10,0
9,5
9,0
8,5
8,0
7,5
7,0
6,5
6,0
0
18
24
42
48
Time (hours)
E138vsSC16
FA18vsSC16


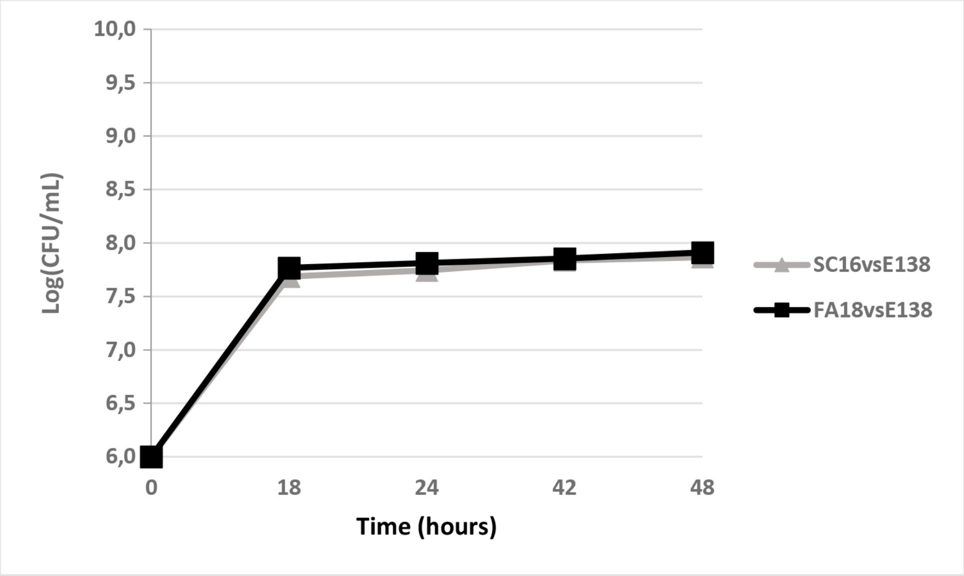

Log(CFU/mL)
10,0
9,5
9,0
8,5
8,0
7,5
7,0
6,5
6,0
0
18
24
42
48
Time (hours)
SC16vsE138
FA18vsE138

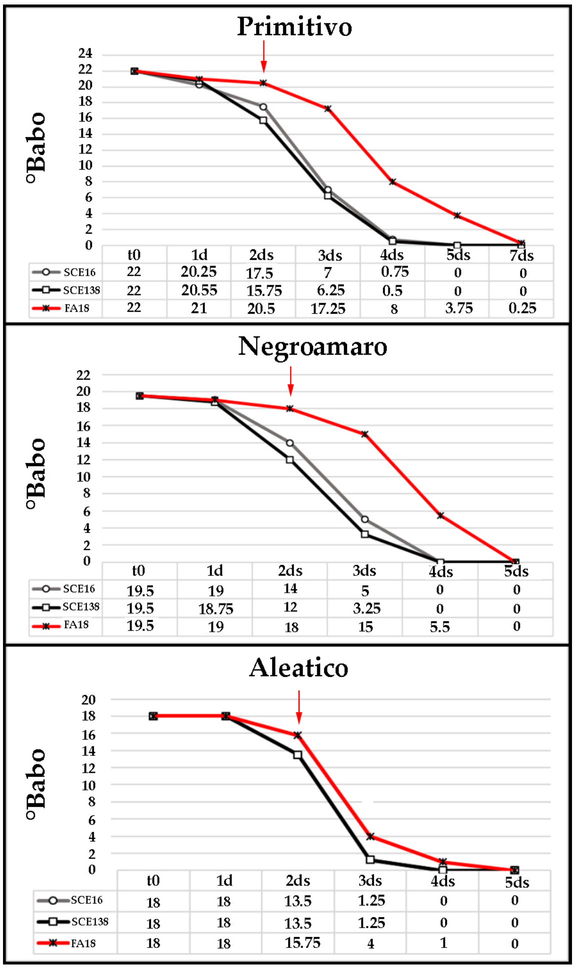


**Primitivo**

| | t0 | 1d | 2ds | 3ds | 4ds | 5ds | 7ds |
|---|---|---|---|---|---|---|---|
| SCE16 | 22 | 20.25 | 17.5 | 7 | 0.75 | 0 | 0 |
| SCE138 | 22 | 20.55 | 15.75 | 6.25 | 0.5 | 0 | 0 |
| FA18 | 22 | 21 | 20.5 | 17.25 | 8 | 3.75 | 0.25 |

**Negroamaro**

| | t0 | 1d | 2ds | 3ds | 4ds | 5ds |
|---|---|---|---|---|---|---|
| SCE16 | 19.5 | 19 | 14 | 5 | 0 | 0 |
| SCE138 | 19.5 | 18.75 | 12 | 3.25 | 0 | 0 |
| FA18 | 19.5 | 19 | 18 | 15 | 5.5 | 0 |

**Aleatico**

| | t0 | 1d | 2ds | 3ds | 4ds | 5ds |
|---|---|---|---|---|---|---|
| SCE16 | 18 | 18 | 13.5 | 1.25 | 0 | 0 |
| SCE138 | 18 | 18 | 13.5 | 1.25 | 0 | 0 |
| FA18 | 18 | 18 | 15.75 | 4 | 1 | 0 |

**Table S1.** Concentration of organic acids in the studied wines.

| | ***Primitivo A*** | | | ***Primitivo B*** | | |
|---|---|---|---|---|---|---|
| | **SCE16** | **SCE138** | **FA18** | **SCE16** | **SCE138** | **FA18** |
| **Tartaric acid** (g/L) | 2.8 ± 0.4 | 2.8 ± 0.5 | 2.8 ± 0.5 | 2.7 ± 0.3 | 2.7 ± 0.4 | 2.8 ± 0.4 |
| **Malic acid** (g/L) | 1.36 ± 0.18 | 1.37 ± 0.15 | 1.38 ± 0.15 | 1.33 ± 0.17 | 1.35 ± 0.17 | 1.36 ± 0.15 |
| **Lactic acid** (g/L) | 0.113 ± 0.017 | 0.11 ± 0.02 | 0.12 ± 0.02 | 0.112 ± 0.015 | 0.113 ± 0.016 | 0.113 ± 0.017 |
| **Citric acid** (g/L) | 0.24 ± 0.05 | 0.23 ± 0.04 | 0.24 ± 0.04 | 0.25 ± 0.03 | 0.24 ± 0.03 | 0.24 ± 0.05 |
| | *Negramaro A* | | | *Negramaro B* | | |
| | **SCE16** | **SCE138** | **FA18** | **SCE16** | **SCE138** | **FA18** |
| **Tartaric acid** (g/L) | 2.3 ± 0.3 | 2.3 ± 0.4 | 2.4 ± 0.4 | 2.1 ± 0.3 | 2.2 ± 0.3 | 2.3 ± 0.3 |
| **Malic acid** (g/L) | 1.48 ± 0.19 | 1.49 ± 0.16 | 1.54 ± 0.17 | 1.39 ± 0.18 | 1.46 ± 0.19 | 1.51 ± 0.18 |
| **Lactic acid** (g/L) | 0.055 ± 0.008 | 0.056 ± 0.010 | 0.058 ± 0.010 | 0.052 ± 0.007 | 0.055 ± 0.008 | 0.057 ± 0.009 |
| **Citric acid** (g/L) | 0.33 ± 0.06 | 0.33 ± 0.06 | 0.35 ± 0.06 | 0.31 ± 0.04 | 0.33 ± 0.05 | 0.34 ± 0.06 |
| | *Aleatico A* | | | *Aleatico B* | | |
| | **SCE16** | **SCE138** | **FA18** | **SCE16** | **SCE138** | **FA18** |
| **Tartaric acid** (g/L) | 2.1 ± 0.3 | 2.1 ± 0.4 | 2.3 ± 0.4 | 2.0 ± 0.2 | 2.0 ± 0.3 | 2.3 ± 0.3 |
| **Malic acid** (g/L) | 1.37 ± 0.18 | 1.36 ± 0.15 | 1.48 ± 0.16 | 1.34 ± 0.17 | 1.35 ± 0.18 | 1.47 ± 0.16 |
| **Lactic acid** (g/L) | 0.051 ± 0.008 | 0.052 ± 0.009 | 0.056 ± 0.010 | 0.050 ± 0.007 | 0.048 ± 0.007 | 0.055 ± 0.008 |
| **Citric acid** (g/L) | 0.31 ± 0.06 | 0.30 ± 0.05 | 0.33 ± 0.06 | 0.28 ± 0.07 | 0.30 ± 0.04 | 0.34 ± 0.06 |

**Table S2**. Quantities of the identified anthocyanins into wines. n.d: not detected or under the detection threshold.

| **Compound** | **Primitivo** | | | | | |
|---|---|---|---|---|---|---|
| | ***Draining off*** | | | ***18 months aging*** | | |
| | ***FA18*** | ***SCE16*** | ***SCE138*** | ***FA18*** | ***SCE16*** | ***SCE138*** |
| (epi)-catechin-peonidin 3O-glucoside | 1.4±0.1 | 2.2±0.1 | 1.8±0.4 | 1.3±0.5 | 2.5±0.8 | 1.9±0.2 |
| (epi)-catechin-malvidin 3O-glucoside | 5.0±0.3 | 5.6±0.0 | 5.1±0.7 | 6.1±0.5 | 7.4±0.6 | 6.2±1.3 |
| delphinidin 3O-glucoside | 53.9±2.9 | 63.3±2.1 | 58.0±2.1 | 45.0±0.7 | 62.3±2.7 | 44.4±11.7 |
| di(epi)catechin-malvidin 3O-glucoside | 4.2±0.5 | 3.2±0.7 | 2.5±0.8 | 5.7±0.1 | 4.2±0.3 | 3.9±0.4 |
| cyanidin 3O-glucoside | 14.1±0.8 | 11.2±0.5 | 9.9±0.1 | 12.8±0.5 | 11.8±0.8 | 8.2±3.3 |
| petunidin 3O-glucoside | 109.8±4.3 | 134.1±4.3 | 128.3±5.2 | 93.5±2.9 | 132.1±7.4 | 99.7±25.6 |
| petunidin 3O-glucoside pyruvic derivative | 4.2±0.2 | 6.3±0.1 | 5.8±0.2 | 7.8±0.0 | 10.2±0.2 | 8.2±1.8 |
| peonidin 3O-glucoside | 97.1±6.9 | 99.5±2.5 | 87.0±10.6 | 78.4±0.0 | 92.4±3.6 | 65.4±21.1 |
| malvidin 3O-glucoside | 973.2±65.2 | 1180.8±20.9 | 1113.7±87.5 | 788.7±10.3 | 1127.9±48.5 | 839.1±261.9 |
| malvidin 3O-glucoside pyruvic derivative | 24.3±0.6 | 14.3±0.5 | 12.2±1.9 | 30.8±0.2 | 16.2±0.0 | 14.9±0.8 |
| malvidin 3O-glucoside-8-ethyl-(epi)catechin | 13.1±3.5 | 12.6±3.7 | 5.4±1.4 | 11.1±6.6 | 4.4±0.9 | 3.9±0.2 |
| malvidin 3O-glucoside-8-ethyl-(epi)catechin | 4.5±0.3 | 2.6±1.2 | 1.7±0.6 | 3.6±3.1 | nd | nd |
| peonidin 3O-(*p*-coumaryl)-glucoside pyruvic derivative | 6.0±0.6 | 6.6±0.0 | 5.9±1.3 | 5.6±0.5 | 8.6±0.1 | 5.8±1.8 |
| peonidin 3O-acetylglucoside<br>malvidin 3O-(*p*-coumaryl)-glucoside pyruvic derivative | 4.1±0.4 | 4.8±0.4 | 4.6±0.4 | 3.8±0.2 | 5.6±0.3 | 4.0±1.4 |
| malvidin 3O-acetylglucoside | 71.0±9.1 | 83.7±2.4 | 75.0±9.1 | 62.4±0.7 | 83.4±3.1 | 60.8±20.7 |
| malvidin 3O-acetyl-4-vinyl-(epi)catechin | 3.2±0.4 | 4.5±1.3 | 6.5±1.1 | 3.6±1.9 | 5.6±0.3 | 5.5±1.8 |
| malvidin 3O-caffeoylglucoside<br>cyanidin 3O-(*p*-coumaryl)-glucoside | 12.4±1.2 | 15.2±0.6 | 14.3±1.9 | 8.9±0.3 | 16.2±0.7 | 11.4±3.2 |
| petunidin 3O-(p-coumaryl)-glucoside<br>malvidin 3O-*cis*-(*p*-coumaryl)-glucoside | 14.4±0.9 | 16.3±0.4 | 14±1.8 | 12.1±2.3 | 15.3±0.1 | 11.2±2.9 |
| peonidin 3O-(*p*-coumaryl)-glucoside-8-ethyl-(epi)catechin | 2.0±0.4 | 1.5±0.1 | 1.4±0.5 | 3.5±0.2 | 2.7±0.4 | 2.2±0.3 |
| peonidin 3O-*trans*-(p-coumaryl)-glucoside | 19.1±2.0 | 24.7±2.2 | 22.1±2.3 | 15.9±0.3 | 24.7±0.2 | 17.1±5.3 |
| malvidin 3O-*trans*-(p-coumaryl)-glucoside | 130.6±13.0 | 167.4±12.4 | 156.0±13.7 | 95.2±16.1 | 161.1±2.9 | 117.7±34.5 |
| malvidin 3O-glucoside-4-vinyl-(epi)catechin | nd | nd | nd | nd | nd | nd |
| malvidin 3O-(*p*-coumaroyl)-glucoside-8-ethyl-(epi)catechin | 2.8±1.2 | 5.0±0.3 | 4.0±1.1 | 3.2±0.8 | 6.8±0.0 | 5.3±0.7 |

| **Compound** | **Negroamaro** | | | | | |
|---|---|---|---|---|---|---|
| | ***Draining off*** | | | ***18 months aging*** | | |
| | ***FA18*** | ***SCE16*** | ***SCE138*** | ***FA18*** | ***SCE16*** | ***SCE138*** |
| (epi)-catechin-peonidin 3O-glucoside | 1.2±0.7 | 1.6±0.4 | 1.3±0.1 | 0.5±0.7 | 2.3±0.1 | nd |
| (epi)-catechin-malvidin 3O-glucoside | 3.1±0.6 | 3.1±0.1 | 2.9±0.4 | 3.0±0.3 | 4.2±0.1 | 3.1±0.6 |
| delphinidin 3O-glucoside | 48.1±6.2 | 65.8±7.3 | 60.6±4.1 | 28.0±4.1 | 56.4±19.6 | 31.5±12.6 |
| di(epi)catechin-malvidin 3O-glucoside | 2.8±0.1 | 1.3±0.3 | 1.0±0.0 | 3.9±0.7 | 2.0±0.0 | 2.6±0.6 |
| cyanidin 3O-glucoside | 5.0±0.1 | 5.4±0.6 | 5.6±0.4 | 3.4±0.3 | 6.7±2.0 | 3.3±2.2 |
| petunidin 3O-glucoside | 126.8±21.9 | 179.8±9.6 | 168.5±4.0 | 77.8±14.1 | 155.9±34.6 | 91.5±29.3 |
| petunidin 3O-glucoside pyruvic derivative | 8.3±0.1 | 9.5±1.1 | 8.4±0.4 | 6.7±0.0 | 8.5±0.8 | 5.7±0.7 |
| peonidin 3O-glucoside | 31.8±2.7 | 31.7±7.5 | 28.9±0.3 | 14.0±1.0 | 22.2±3.7 | 15.2±5.2 |
| malvidin 3O-glucoside | 663.1±100.4 | 854.7±8.0 | 808.0±25.4 | 402.4±60.4 | 727.7±96.9 | 447.2±118.9 |
| malvidin 3O-glucoside pyruvic derivative | 14.1±6.5 | 9.1±4.7 | 5.8±0.3 | 18.9±7.5 | 6.3±0.6 | 9.8±1.6 |
| malvidin 3O-glucoside-8-ethyl-(epi)catechin | nd | nd | nd | nd | nd | nd |
| malvidin 3O-glucoside-8-ethyl-(epi)catechin | nd | nd | nd | nd | nd | nd |
| peonidin 3O-(p-coumaryl)-glucoside pyruvic derivative | 11.7±2.5 | 13.7±1.5 | 12.2±0.2 | 5.2±2.8 | 11.2±3.3 | 6.4±1.3 |
| peonidin 3O-acetylglucoside<br>malvidin 3O-(p-coumaryl)-glucoside pyruvic derivative | 2.5±0.3 | 2.8±0.1 | 3.3±0.2 | 1.3±0.4 | 2.1±0.1 | 0.5±0.7 |
| malvidin 3O-acetylglucoside | 45.9±5.0 | 57.0±1.5 | 53.6±1.0 | 27.8±3.0 | 47.3±7.8 | 29.2±7.4 |
| malvidin 3O-acetyl-4-vinyl-(epi)catechin | nd | nd | nd | nd | nd | nd |
| malvidin 3O-caffeoylglucoside<br>cyanidin 3O-(p-coumaryl)-glucoside | 17.5±4.8 | 21.5±2.3 | 20.7±0.6 | 10.6±4.0 | 18.2±4.7 | 9.7±3.7 |
| petunidin 3O-(p-coumaryl)-glucoside<br>malvidin 3O-cis-(p-coumaryl)-glucoside | 3.7±0.5 | 6.4±3.0 | 3.9±0.0 | 2.1±0.1 | 3.7±0.6 | 2.7±0.6 |
| peonidin 3O-(p-coumaryl)-glucoside-8-ethyl-(epi)catechin | nd | nd | nd | nd | nd | nd |
| peonidin 3O-trans-(p-coumaryl)-glucoside | 8.2±0.4 | 6.4±0.7 | 6.4±0.3 | 8.4±0.4 | 7.6±0.9 | 3.2±1.4 |
| malvidin 3O-trans-(p-coumaryl)-glucoside | 75.1±16.6 | 96.4±5.5 | 87.1±2.0 | 42.5±8.5 | 79.8±19.6 | 44.3±17.2 |
| malvidin 3O-glucoside-4-vinyl-(epi)catechin | nd | nd | nd | 3.0±0.1 | 2.9±1.0 | 1.5±2.1 |
| malvidin 3O-(p-coumaroyl)-glucoside-8-ethyl-(epi)catechin | 23.0±8.7 | 11.2±3.0 | 8.4±1.7 | 28.2±5.7 | 11.5±2.9 | 8.7±1.0 |

n.d: not detected or under the detection threshold.

| **Compound** | **Aleatico** | | | | | |
|---|---|---|---|---|---|---|
| | *Draining off* | | | *18 months aging* | | |
| | **FA18** | **SCE16** | **SCE138** | **FA18** | **SCE16** | **SCE138** |
| (epi)-catechin-peonidin 3O-glucoside | 3.3±0.1 | 3.4±0.6 | 2.6±0.6 | 3.0±1.9 | 3.8±0.3 | 4.8±0.6 |
| (epi)-catechin-malvidin 3O-glucoside | 3.4±0.3 | 4.1±0.2 | 4.2±0.0 | 2.3±1.6 | 4.0±0.5 | 4.0±0.1 |
| delphinidin 3O-glucoside | 85.2±1.4 | 105.4±26.5 | 105.6±11.0 | 61.2±0.4 | 90.1±18.9 | 89.4±25.8 |
| di(epi)catechin-malvidin 3O-glucoside | 1.1±0.3 | 2.7±0.4 | 2.8±0.4 | 2.0±0.6 | 1.1±0.1 | 1.1±0.1 |
| cyanidin 3O-glucoside | 30.1±0.0 | 42.2±12.5 | 40.7±6.6 | 24.6±0.9 | 39.7±7.1 | 38.4±10.7 |
| petunidin 3O-glucoside | 169.9±6.2 | 197.0±39.7 | 201.9±15.1 | 121.5±0.7 | 172.1±26.1 | 173.5±45.5 |
| petunidin 3O-glucoside pyruvic derivative | 7.9±0.1 | 7.6±0.8 | 7.7±0.2 | 6.7±0.0 | 7.2±0.6 | 7.4±0.5 |
| peonidin 3O-glucoside | 77.1±2.2 | 110.5±19.3 | 112.0±9.9 | 55.1±1.3 | 94.0±15.1 | 94.4±28.0 |
| malvidin 3O-glucoside | 585.5±25.1 | 654.7±99.3 | 703.6±46.9 | 409.3±1.7 | 562.1±61.0 | 589.6±153.7 |
| malvidin 3O-glucoside pyruvic derivative | 7.0±1.0 | 5.3±0.3 | 5.6±0.4 | 9.2±1.0 | 6.6±0.1 | 6.8±1.7 |
| malvidin 3O-glucoside-8-ethyl-(epi)catechin | nd | nd | nd | nd | nd | nd |
| malvidin 3O-glucoside-8-ethyl-(epi)catechin | nd | nd | nd | nd | nd | nd |
| peonidin 3O-(p-coumaryl)-glucoside pyruvic derivative | nd | nd | nd | nd | nd | nd |
| peonidin 3O-acetylglucoside<br>malvidin 3O-(p-coumaryl)-glucoside pyruvic derivative | nd | nd | nd | nd | nd | nd |
| malvidin 3O-acetylglucoside | 3.4±0.0 | 3.5±0.7 | 3.4±0.1 | 3.0±0.6 | 2.9±0.3 | 3.1±0.8 |
| malvidin 3O-acetyl-4-vinyl-(epi)catechin | nd | nd | nd | nd | nd | nd |
| malvidin 3O-caffeoylglucoside<br>cyanidin 3O-(p-coumaryl)-glucoside | 1.6±0.1 | 1.3±0.4 | 1.5±0.1 | 1.6±0.1 | 1.2±0.1 | 1.1±0.3 |
| petunidin 3O-(p-coumaryl)-glucoside<br>malvidin 3O-cis-(p-coumaryl)-glucoside | nd | nd | nd | nd | nd | nd |
| peonidin 3O-(p-coumaryl)-glucoside-8-ethyl-(epi)catechin | nd | nd | nd | nd | nd | nd |
| peonidin 3O-trans-(p-coumaryl)-glucoside | 5.0±0.3 | 3.8±0.6 | 4.4±0.0 | 4.7±0.9 | 3.6±0.2 | 3.1±0.1 |
| malvidin 3O-trans-(p-coumaryl)-glucoside | 4.1±2.6 | 6.8±1.4 | 6.1±0.9 | 2.8±0.8 | 3.5±4.9 | 0.7±1.0 |
| malvidin 3O-glucoside-4-vinyl-(epi)catechin | nd | nd | nd | nd | nd | nd |
| malvidin 3O-(p-coumaroyl)-glucoside-8-ethyl-(epi)catechin | 8.4±1.1 | 4.0±0.0 | 4.3±0.5 | 13.7±7.2 | 4.0±0.4 | 5.0±1.0 |
